# Genome-wide meta-analysis of maize heterosis reveals the potential role of additive gene expression at pericentromeric loci

**DOI:** 10.1186/1471-2229-14-88

**Published:** 2014-04-02

**Authors:** Alexander Thiemann, Junjie Fu, Felix Seifert, Robert T Grant-Downton, Tobias A Schrag, Heike Pospisil, Matthias Frisch, Albrecht E Melchinger, Stefan Scholten

**Affiliations:** 1 Biocenter Klein Flottbek, Developmental Biology and Biotechnology, University of Hamburg, Hamburg 22609, Germany; 2 Institute of Crop Sciences, Chinese Academy of Agricultural Sciences, Beijing 100081, PR China; 3 Department of Plant Sciences, University of Oxford, Oxford OX1 3RB, UK; 4 Institute for Plant Breeding, Seed Science and Population Genetics, Applied Genetics and Plant Breeding, University of Hohenheim, Stuttgart 70599, Germany; 5 Department of Bioinformatics, Technical University of Applied Sciences Wildau, Wildau 15745, Germany; 6 Institute of Agronomy and Plant Breeding II, Biometry and Population Genetics, Justus-Liebig University, Giessen 35392, Germany; 7 Institute for Plant Breeding, Seed Science and Population Genetics, Plant Breeding and Biotechnology, University of Hohenheim, Stuttgart 70599, Germany

**Keywords:** Heterosis, Maize, QTL, Grain yield, Additive gene expression, Haplotype

## Abstract

**Background:**

The identification of QTL involved in heterosis formation is one approach to unravel the not yet fully understood genetic basis of heterosis - the improved agronomic performance of hybrid F1 plants compared to their inbred parents. The identification of candidate genes underlying a QTL is important both for developing markers and determining the molecular genetic basis of a trait, but remains difficult owing to the large number of genes often contained within individual QTL. To address this problem in heterosis analysis, we applied a meta-analysis strategy for grain yield (GY) of *Zea mays* L. as example, incorporating QTL-, hybrid field-, and parental gene expression data.

**Results:**

For the identification of genes underlying known heterotic QTL, we made use of tight associations between gene expression pattern and the trait of interest, identified by correlation analyses. Using this approach genes strongly associated with heterosis for GY were discovered to be clustered in pericentromeric regions of the complex maize genome. This suggests that expression differences of sequences in recombination-suppressed regions are important in the establishment of heterosis for GY in F1 hybrids and also in the conservation of heterosis for GY across genotypes. Importantly functional analysis of heterosis-associated genes from these genomic regions revealed over-representation of a number of functional classes, identifying key processes contributing to heterosis for GY. Based on the finding that the majority of the analyzed heterosis-associated genes were addtitively expressed, we propose a model referring to the influence of cis-regulatory variation on heterosis for GY by the compensation of fixed detrimental expression levels in parents.

**Conclusions:**

The study highlights the utility of a meta-analysis approach that integrates phenotypic and multi-level molecular data to unravel complex traits in plants. It provides prospects for the identification of genes relevant for QTL, and also suggests a model for the potential role of additive expression in the formation and conservation of heterosis for GY via dominant, multigenic quantitative trait loci. Our findings contribute to a deeper understanding of the multifactorial phenomenon of heterosis, and thus to the breeding of new high yielding varieties.

## Background

Heterosis (hybrid vigor), the improved performance of F_1_ hybrid plants compared with their inbred parents, has been used by plant breeders to develop crosses with improved agronomic characteristics for many years [1,2]. The biological phenomenon heterosis is described by the trait-specific performance of hybrids relative to the average of its two parents, termed mid-parent heterosis (MPH) or relative to the parent having the best value for the trait, termed best-parent heterosis (BPH).

In breeding, QTL mapping is used to identify chromosomal regions contributing to agronomic traits [3] and integration of data from different populations has led to the identification of QTL associated with hybrid performance (HP) and heterosis in maize [4,5]. The identification of candidate genes underlying a QTL is important both for developing markers and determining the molecular genetic basis of a trait, but remains difficult to achieve owing to the large number of genes often contained within individual QTL [6]. Data on gene expression have been used to identify candidate genes in animals [7], for review] and have also been employed in the search for genes involved in water-use efficiency in cereals [8]. The combination of QTL mapping data and gene expression analyses has led to the identification of candidate genes regulating grain fiber content in wheat [9] and grain weight in maize [10]. Despite the merits of this approach, the large size of many QTL, the number of genes within them and particularly their loose association with both locus and trait makes assigning individual genes or groups of genes to particular traits very challenging.

We have addressed this specific problem for heterosis for GY - an important heterotic trait by carrying out gene/QTL co-localization analyses only where a close relationship with heterosis had been observed. For genes, this relationship was based on expression pattern such that they showed differential expression levels in the parents, and their additive, mid-parental expression level significantly correlated with MPH for GY [11]. The heterotic QTL we used have been demonstrated to exhibit certain conservation between three maize populations [4]. To combine these different datasets by a meta-analysis, the genomic positions of QTL and the genes identified from different maize lines were projected onto a physical map of the B73 reference genome. Genomic segments, with an overrepresentation of heterosis for GY-correlated genes, were subsequently analyzed for co-localization with the heterotic QTL.

This meta-analysis strategy provides a high level of confidence when associating individual genes with QTL and enabled us to determine the identity of genes that are situated at QTL significantly associated with heterosis for GY. We further investigated the functions of these genes and determined functional classes of genes that are enriched at the heterotic loci identified. Finally, we performed microarray analysis of nine hybrids and their corresponding parents to quantify the actual relative expression variation for MPH for GY-correlated genes in hybrids.

## Results

### *In silico* mapping of genes correlated with mid-parental heterosis

**Figure 1:**
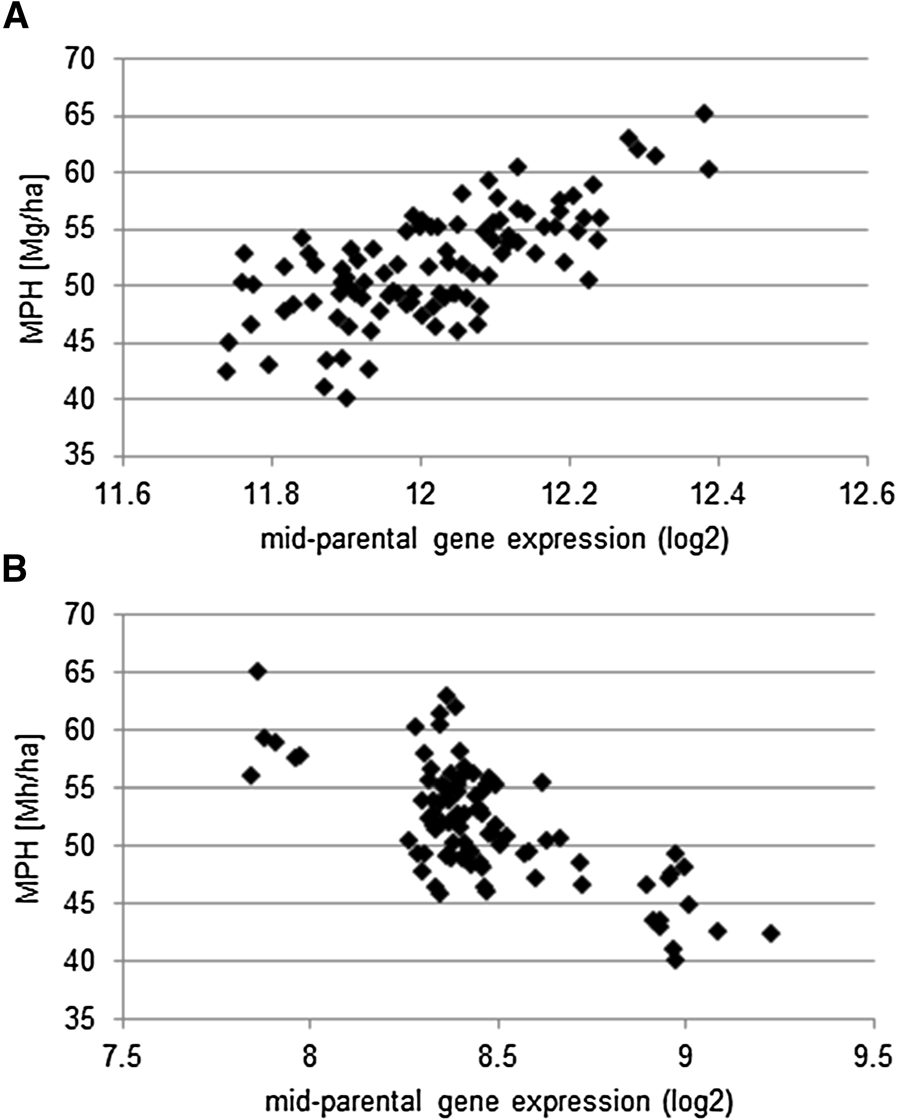
**Linear correlation between calculated additive gene expression levels and MPH for GY hybrid field data.** Shown are MPH for GY field data (y-axis) [Mg/ha] and the calculated additive gene expression levels [(log2(P1) + log2(P2))/2] (x-axis) of the 98 hybrids for two genes. **A**: Gene MZ00024213 shows a significantly (p = 7.00E-11) positive correlation (r = 0.68) with MPH for GY and **B**: MZ00022903 shows a significantly (p = 7.52E-11) negative correlation (r = −0.67) with MPH for GY.

Our meta-analysis for the identification of QTL-associated genes relies on establishing strong associations between genetic expression levels and the trait of interest. We therefore analyzed genes with a differential parental expression and a linear correlation (Pearson product–moment correlation, p ≤ 0.01) between the additive, mid-parental gene expression levels and MPH for GY field data from Thiemann *et al.*[11]. The field data (14 Dent and 7 Flint parental inbred lines) comprised four Flint lines with an European Flint background three with an Flint/Lancaster background, eight Dent lines with an Iowa Stiff Stalk Synthetic background and six Dent lines with an Iodent background and 98 corresponding hybrids (field data in Additional file 1), which were combined in a mixed model analysis with field data of additional factorials obtaining best linear unbiased predictors (BLUP) [12,13]. Two examples of a positively and a negatively MPH for GY-correlated gene are shown in Figure 1.

If MPH-correlated genes contribute significantly to heterosis, this role should be interpretable in terms of quantitative genetics. To this end, we analyzed the 1,999 MPH-correlated genes from Thiemann *et al.*[11] for possible co-localization with known heterotic QTL for GY, by first mapping those genes to the B73 maize genome followed by distribution analysis.

We compared the genome locations of the MPH-correlated genes, *in silico*, to a random gene distribution reflecting the actual distribution of genes in the B73 genome, to discover whether the MPH-correlated genes are distributed randomly or non-randomly in the maize genome. A non-random distribution of the MPH-correlated genes would thus indicate a level of functional grouping. The random gene distribution was calculated via bootstrapping, in which random sets of oligonucleotides from the 46k-microarray (microarray originally used for the identification of the 1,999 MPH-correlated genes [11]; GEO Platform accession number GPL6438) and of the subset size of the mapped MPH-correlated genes were mapped to 145 equally sized genomic segments (see Additional file 2).

**Figure 2:**
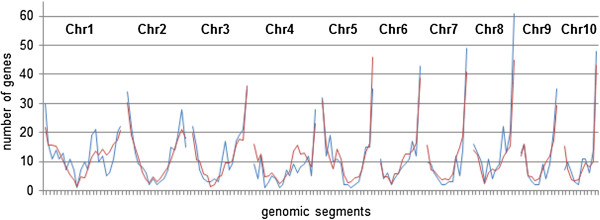
**Gene distribution of MPH-correlated genes across the ten chromosomes of the maize genome (B73).** The figure shows the number of MPH-correlated genes per genomic segment (blue lines). The red lines show the average number of random genes per genomic segment as calculated from bootstrap analysis. Significant difference between the two gene distributions was shown by a chi-square test (*χ*^2^ (df = 144, n = 1654) = 183.84, p = 0.014).

**Figure 3:**
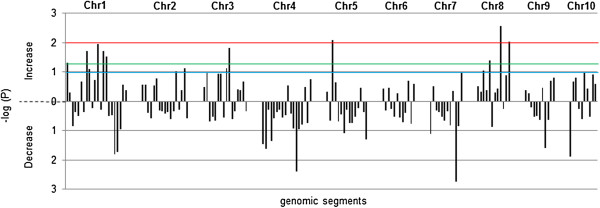
**Negative decadic logarithms of p-values from bootstrap analysis of the 145 genomic segments across chromosomes.** Negative decadic logarithms of p-values (−log(P)) from the bootstrap analysis show the significance in the increase and decrease of MPH-associated gene numbers compared to the gene number of the random gene distribution. Thresholds of 1 (blue line, p = 0.1), 1.3 (green line, p = 0.05) and 2 (red line, p = 0.01) were chosen for the identification of MPH-ASs.

In total 1,654 (82.74%) of the 1,999 MPH-correlated genes were unambiguously localized on the B73 genome by mapping and filtering analysis of the array probe sequences. These genes were located on all 10 maize chromosomes in a non-random pattern that diverged significantly (*χ*^2^ (df = 144, n = 1654) = 183.84; p = 0.014) from the random gene distribution (Figure 2). To identify the genomic regions contributing most to the overall significant differences, we focused on regions of the genome containing a significantly elevated number of MPH for GY-correlated genes. We termed these segments MPH-associated genomic segments (MPH-ASs). Thus MPH-ASs with a threshold p-value of 0.1 in the bootstrap analysis indicated an increased number of MPH-associated genes compared to the expected gene number per genomic segment, ranging from 1.32 to 2.27 fold. In total, we identified 15 MPH-ASs containing a total of 290 MPH-correlated genes (Additional file 3). The three most significant MPH-ASs (p-value below 0.01 in the bootstrap assay) are located on chromosomes 5 and 8. Additional MPH-ASs with p-values below 0.05 are located on chromosomes 1, 3 and 8. Five MPH-ASs with p-values below 0.1 are located on chromosomes 1, 2, 3 and 8. The p-values from the bootstrap analysis of the individual segments are represented as negative decadic logarithms in Figure 3.

**Figure 4:**
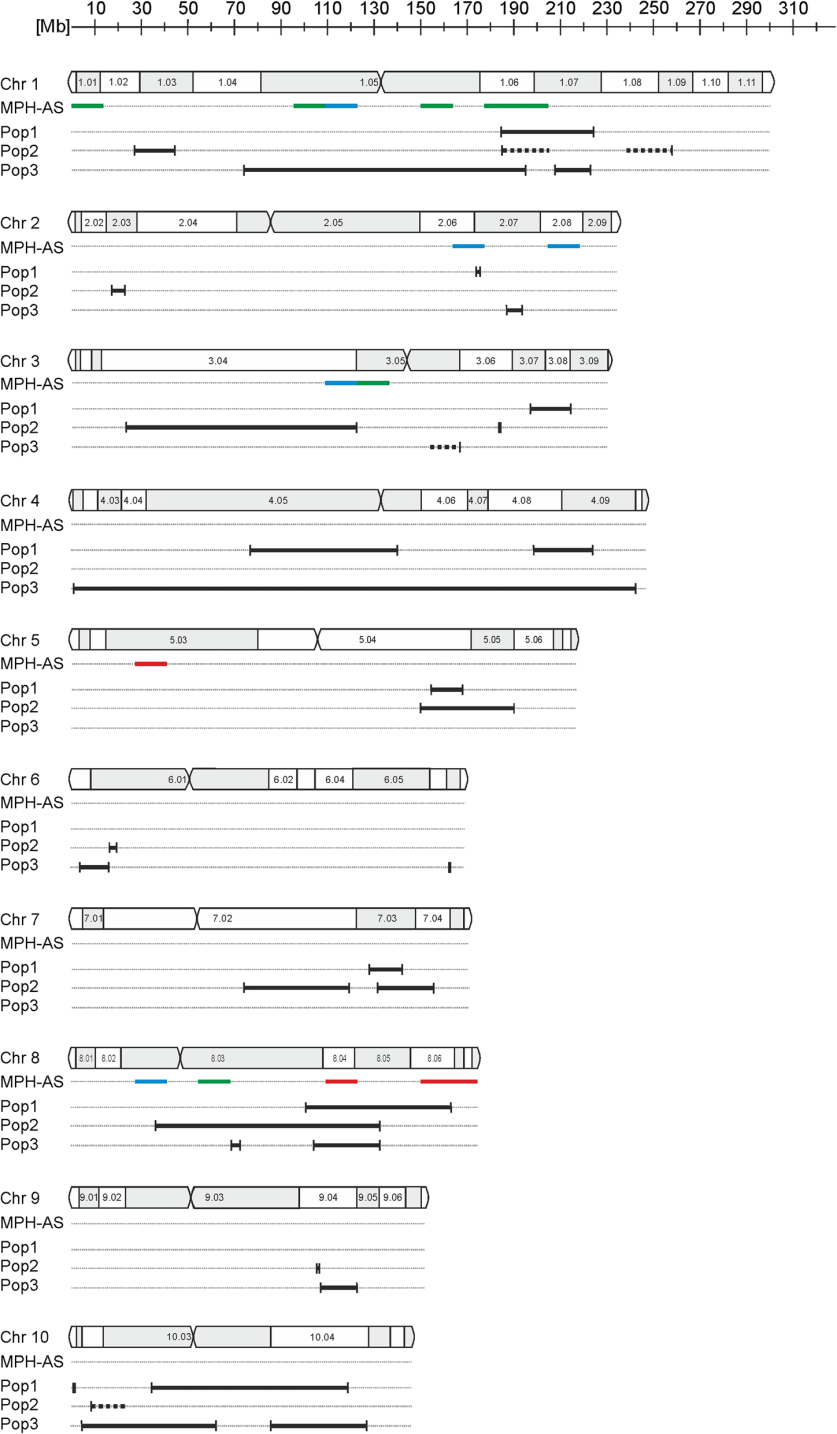
**Co-localization of the MPH-ASs with heterotic QTLs for GY.** Co-localization of the identified MPH-ASs with all heterotic QTL for GY with significant dominance effect from three populations re-analyzed by Schön *et al.*[4] (black lines) on all ten maize chromosomes (accurate scale). The MPH-ASs are represented in three different colors, according to the significance (p < 0.01 (red lines); 0.01 < p < 0.05 (green lines); 0.05 < p < 0.1 (blue lines)) of their increase in gene number, compared to the random gene distribution during bootstrapping. The three populations (Pop) re-analyzed by Schön *et al.*[4] are: Pop1 from Stuber *et al.*[14], Pop2 from Lu *et al.*[15] and Pop3 from Frascaroli *et al.*[16]. The vertical lines of the QTL are representing the genetic markers flanking the confidence interval of the QTL. Dashed lines represent QTL with one genetic marker with an unknown physical position. The maize chromosomes are shown with bins, based on core markers from the IBM2 2008 neighbor map.

Surprisingly, mapping these MPH-ASs to the B73 genome revealed a predominant localization close to centromeres. Of the 15 MPH-ASs, six locate inside bins comprising the centromere, and six are located in bins adjacent to centromeres (Figure 4). Schön *et al.*[4] proposed that parental alleles involved in heterosis became fixed adjacent to centromeres as a result of the low recombination rates in these regions. Such a fixation of alleles could result in higher levels of differential expression. To investigate this further we analyzed the frequency of differential expression of all mapped MPH-correlated genes between pairs of the 21 inbred lines of the original breeding factorial [11]. We found that the 290 MPH-correlated genes contained within the MPH-ASs were differentially expressed between more pairs of inbred lines than the residual MPH-correlated genes (two-sided *t*-test, t-value = 2,735, df = 1997, p = 0.006, see Additional file 4).

### Co-localization of genes with known heterotic QTL

QTL co-localization of the 1,999 MPH-correlated genes was performed with heterotic QTL for GY from Schön *et al.*[4], who re-analyzed QTL from three different maize populations with a Stiff Stalk Synthetic and Lancaster genetic background [14-16] by performing two joint fit linear transformations. One of the two transformations, named Z_2_ (half the trait difference between pairs of backcross progenies), addresses the augmented dominance effects (described in Melchinger *et al.*[17]).

In comparison to QTL data from Schön *et al.*[4], 11 of the 15 MPH-ASs were shown to co-localize with at least one heterotic QTL for GY, with an augmented dominance effect (Z_2_) (Figure 4, see Additional file 5). Schön *et al.*[4] identified heterotic Z_2_ QTL solely present in one or two of the three different maize populations as well as QTL with a congruent genomic position between all populations. These conserved, congruent QTL were identified on three genomic positions on chromosome 1, 8 and 10. Most of our MPH-ASs are located on chromosomes 1 and 8, and co-localize with the congruent QTL positions described by Schön *et al.*[4]. Specifically, two MPH-ASs co-localize with all three QTL at the congruent genomic position on chromosome 1, and three MPH-ASs co-localize with one of the three QTL. On chromosome 8 all four MPH-ASs co-localize with at least one of the three congruent QTL described by Schön *et al.*[4]. The MPH-AS with the most significant number of MPH-correlated genes (MPH-AS8.9) co-localizes with all three congruent QTL on chromosome 8. Furthermore, MPH-ASs on chromosomes 2 and 3 were found to co-localize with less conserved heterotic QTL found in solely one population analyzed by Schön *et al.*[4].

To test the significance of co-localization of the MPH-ASs with the heterotic QTL described by Schön *et al.*[4], chi-square analysis was carried out comparing the QTL coverage of the B73 genome (45.84%) and the MPH-ASs coverage of the QTL. In total 128,441,964 bp (59.62%) of the MPH-ASs co-localize with the heterotic QTL, resulting in significant p-values (p ≤ 0.01) and confirming that MPH-ASs are significantly associated with the heterotic QTL described by Schön *et al.*[4].

We investigated the possibility whether absolute numbers of MPH-associated genes are a factor influencing QTL co-localization, by counting them on all genomic segments, with (average gene number = 11.5, SD = 10.82, SE = 1.17) and without (average gene number = 11.27, SD = 9.1, SE = 1.2) co-localization. The data showed that gene number alone was not a significant factor (two-sided *t*-test, t-value = 0.134, df = 143, p = 0.89) in the co-localization of segments with any of the heterotic QTL described by Schön *et al.*[4].

### Functional characterization of genes contributing to heterosis

To determine the biological functions of the 290 MPH-correlated genes located on MPH-ASs (see Additional file 6), they were sorted into functional categories using the MIPS Functional Catalogue Database (FunCatDB, [18]). In this database more than one functional category can be assigned to an individual gene.

**Figure 5:**
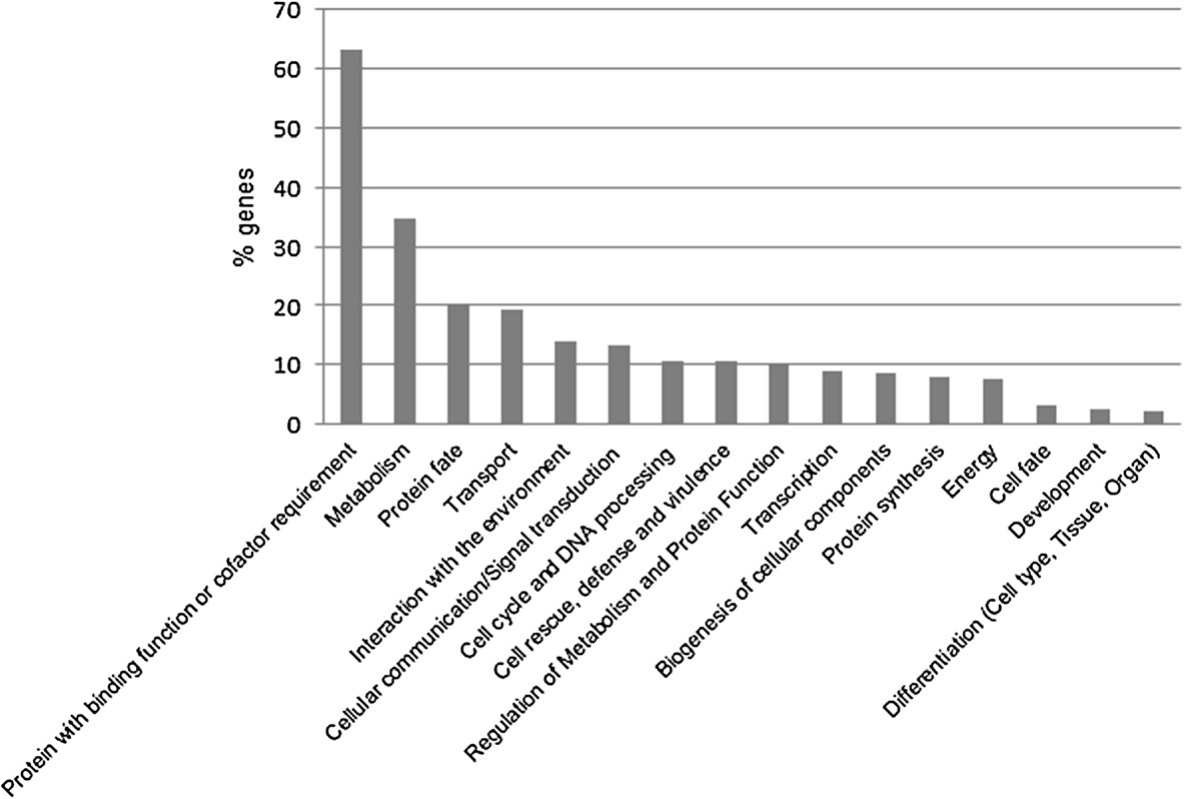
**Functional classifications of the MPH-correlated genes, located on the MPH-ASs.** The MPH-correlated genes are, based on their GO annotation, sorted into 16 functional categories, established by the Munich Information Center for Protein Sequences (MIPS; Ruepp *et al.*[18]).

MIPS functional categories were successfully assigned to 188 of the 290 genes (Figure 5). The majority of genes code for proteins with either a binding function or a cofactor requirement (63.3%). The principal biological functions identified were metabolic processes (34.57%), processes related to protein fate (20.21%) including folding, modification and destination of proteins, and processes involved in transport (19.15%). A further significant group of proteins is involved in interaction with the environment (13.83%) and cellular communication and signal transduction (13.3%). Smaller subsets of proteins are indicated to function in cell cycle and DNA processing (10.64%), cell rescue, defense and virulence (10.64%) and the regulation of metabolism and protein function (10.11%).

To explore specific biological processes enriched among genes underlying heterosis for GY, we tested for overrepresentation of GO terms among the 290 MPH-correlated genes located on the MPH-ASs (see Additional file 7). We found significant (p ≤ 0.05) overrepresentation of processes including interaction with the environment (cold acclimation, response to symbiotic fungus, non-photochemical quenching, response to lead ion), protein fate and metabolism (transcription from RNA polymerase I promoter, peptidyl-arginine methylation, amino sugar metabolic process, oligopeptide transport), signal transduction (small GTPase mediated signal transduction), DNA replication and endoreduplication (DNA-dependent DNA replication initiation, regulation of DNA repair, positive regulation of endoreduplication). Additionally, a large subset of genes encoded proteins identified as being involved in transport processes (see Additional file 7).

### Analyzed heterosis-correlated genes are mainly additively expressed

The hybrid expression of the MPH-correlated genes from our former study [11] has not yet been analyzed. For this reason we performed microarray hybridization on a subset of the 1,999 MPH-correlated genes. The custom-made 1.5k-microarray used, comprises 345 MPH-correlated and 174 non-correlated genes (for detailed selection procedure see Methods) and we performed the analysis on nine hybrid genotypes and their corresponding inbred parents. The F1 hybrid combinations analyzed were selected to cover a wide range of MPH levels for GY (see Additional file 8).

We first identified genes differentially expressed in the nine hybrid-inbred triplets (i.e. two parental inbred lines and their hybrid combination), and found 14.2% (49 of 345) of the MPH-correlated genes and 12.07% (21 of 174) of the non-correlated genes to be differentially expressed in at least one of the nine hybrid-inbred triplets (see Additional file 9).

To validate these microarray results, the data were compared with the 46k-microarray data from our previous study [11]. This was possible, since seven inbred lines (P033, P048, S028, S036, F039, F047, L024) were analyzed in both independent studies. In total 63.6% of all 520 genes, including also the non-differential genes, and 92.6% of the significantly (FDR 0.15) differentially expressed genes between the seven inbred lines showed a similar expression tendency in both datasets.

Within the MPH-correlated and the non-correlated subsets, we analyzed the hybrid expression levels of differentially expressed genes and found that, 24.49% (12 of 49) correlated, and 38.1% (8 of 21) non-correlated genes exhibited non-additive expression in at least one hybrid. The remainder of the differentially expressed genes showed a continuum of additive, mid-parental expression pattern among the hybrid-inbred triplets (see Additional file 10).

We then considered the expression of each gene in individual hybrids, and discovered the non-additive expression pattern to appear inconsistently. For example, among the genes with a differential expression in at least one hybrid-inbred triplet no gene was identified with a non-additive expression in a second hybrid. Again, in the remaining hybrids these genes are either additively or not differentially expressed. The low level and stochastic nature of non-additive expression of differentially expressed genes results in an overall proportion of additive expression of 83.1% (with 16.9% non-additive) for MPH-correlated genes, and a slightly smaller contingent of 74.2% (with 25.8% non-additive) for those non-correlated genes.

Further analysis of the non-additively expressed MPH-correlated genes revealed predominantly expression levels similar to one of the two parents, or within the parental range, with only a minor fraction falling outside the parental range (Table 1).

**Table 1:** Expression analyses results of the microarray experiments

							**Non-additive expression pattern**				
	**Genes [no.]**	**Diff. genes [no.]**	**Diff. genes [%]**	**Additive gene expression pattern [%]**	**Non-add. genes [no.]**	**Non-add. genes (of all diff. genes) [%]**	**HPL [%]**	**LPL [%]**	**AHP [%]**	**BLP [%]**	**Between [%]**
Non-correlated	174	21	12.07	74.19	8	38.1	41.7	25	16.7	8.3	8.3
MPH-correlated	345	49	14.2	83.1	12	24.49	8.3	25	0	33.3	0

Shown are the microarray expression results of the MPH for GY-correlated and non-correlated genes. The results consist of the number [no.] and percentage [%] of differentially expressed genes (diff. genes). The percentages of additive gene expression pattern relative to all differential gene expression pattern are given. From the differentially expressed genes the number [no.] and percentage [%] of genes showing a non-additively expression in at least one inbred-hybrid are given. Also the percentages of different patterns of non-additive expression (HPL: high parent like; LPL: low parent like; AHP: above high parent; BLP: below low parent; Between: between both parents) are given.

## Discussion

### Genes contributing to heterosis for GY have a distinct and conserved distribution, even in divergent maize inbreds

Our data show MPH-correlated genes to be distributed in a non-random pattern within the maize genome, with genes involved in heterosis being significantly clustered. This clustering analysis revealed a striking enrichment in centromeric regions. A number of studies have shown that QTL for GY harbor predominantly major dominance or overdominance effects [4,5,19] – effects, which are consistent with multiple MPH-correlated genes being localized in a single QTL. Our results suggest that the individual effects on heterosis of the many MPH-correlated genes underpinning the QTL can result in its overall dominance or overdominance, an inference supported by Charlesworth and Willis [20] who also assumed that linked genes with a small, cumulative phenotypic effect underlie major QTLs, especially in regions with high gene density relative to recombination frequency. Both Larièpe *et al.*[5] and Schön *et al.*[4] assumed fixed parental alleles in regions of low recombination flanking centromeres [21] interact with, or compensate each other to contribute to heterosis; importantly our data confirm these regions to be enriched in heterosis-correlated genes, and reveal thereby the existence of conserved, heterosis-associated genomic regions.

Different maize genomes are characterized by high sequence diversity and broken co-linearity [22,23]. This leads to variation in QTL locations between different mapping populations, and can effectively prevent comparison of genomic features between different maize inbred lines. The fact that we can co-localize MPH-ASs underlying genes with the same QTL in different lines, despite these limitations, reveals the strength of the drivers behind the conservation of these gene clusters. A significant factor contributing to this conservation is likely to be the fixation of allelic *cis*-regulatory elements. These elements are, rather than trans-regulatory elements, sufficient to explain the differential parental expression of additive genes and therefore, according to our microarray results, for the majority of MPH-ASs underlying genes in the pericentromeric regions of the 21 inbred lines studied.

The higher rates of conserved differential parental expression observed, also suggest that centromeric regions are diverging rapidly at the sequence level and that initially this can result in hybrid vigor. This fits with the model of “meiotic drift”, in which selfish sequences diverge rapidly and thereby confer a transmission advantage during meiosis [24,25]. For this reason, rapid sequence divergence in heterochromatic/pericentromeric sequences resulting in high levels of heterosis might be anticipated in maize. Moreover our findings of conserved MPH-ASs in centromere-adjacent genomic bins fits with the observation that in maize meiotic drive can be conferred at multiple locations throughout the genome by repetitive DNA containing heterochromatic knobs with potential neocentromeric function [26].

Additionally, the high conservation of MPH-ASs most probably enabled their identification based on enrichment in gene numbers across diverse inbred lines. Interestingly, MPH-correlated genes in segments other than MPH-ASs, failed to contribute to conserved heterotic effects between the 21 inbred lines and the three populations analyzed by Schön *et al.*[4]. Instead, since differential expression in these segments is less conserved, these MPH-correlated genes are more likely to contribute to heterosis within a limited set of inbred lines.

### Functional analysis of heterosis-associated genes reveals processes contributing to the heterotic phenotype

Heterosis in plants is characterized by an increase in biomass (including GY), faster development and enhanced resistance to biotic and abiotic stress [27-29]. Appropriately, our GO overrepresentation analysis of MPH-ASs underlying MPH-correlated genes revealed significant enrichment in sequences involved in the perception of biotic and abiotic stimuli. These genes have the potential also to contribute to more vigorous development as they enable the plant to react more effectively to environmental stress – an interpretation corroborated by the MIPS analysis, which revealed enrichment in genes involved in interaction with the environment and plant defense. The analysis of the functional classes of our MPH-correlated genes also supports our contention that both dosage effect and epistatic interactions are important in heterosis. A role for epistasis is underlined by the fact that more than 60% of MPH-correlated genes are involved in binding to other proteins or DNA, or acting as co-factors or as components of small GTPase-mediated signal transduction pathways. Likewise, enrichment in signal transduction and metabolism genes lends support to the view that dosage plays an important role in heterosis.

Our work clearly identifies heterosis to be partly a result of the altered expression of a large number of genes encoding proteins involved in a range of biological processes, an interpretation supported by both functional analyses based on MIPS categories and GO overrepresentation analyses. Most importantly, we identified MPH-correlated genes in seven-day-old seedlings, revealing that hybrid expression levels of genes with a measurable impact on MPH for GY are established well before the trait is expressed. While correlations between a number of physiological traits and GY have been shown prior to flowering and seed development [30,31], our data on MPH-correlated genes represent by far the earliest association of this type.

### Additive expression as a component of heterosis

In this study, for a set of MPH-correlated genes, hybrid expression was determined and was shown to be mainly additive (mid-parental). Non-additive expression among the analyzed genes was further shown to be inconsistent among the nine hybrids, all of which exhibited varying, but significant degrees of heterosis. A similar extent of inconsistency has previously been described in maize, with only a small number of genes being non-additively expressed in more than one hybrid [32]. Our findings suggest that the additive, mid-parental expression values used in our previous correlation analysis [11] in most cases accurately reflect the actual expression levels in the 98 hybrids and that, in our experiments and for our analyzed genes, non-additivity does not contribute significantly to heterosis across the genotypes. Additive expression of this type reflects a direct link between parental and hybrid expression levels on account of its mainly exclusive regulation in *cis*[33,34], and explains, at least in part, the effective use of parental transcriptome data to predict MPH [35,36].

Our findings also indicate a quantitative relationship between additive gene expression and heterosis, and are consistent with a positive correlation between percentage additive expression and heterosis reported for maize by Guo *et al.*[27]. That additive gene expression represents a major force in generating heterosis is also supported by the correlation between high genetic variation [37], resulting in predominantly *cis*-regulated additive expression [34,38], and exceptionally high heterosis values in maize [39].

### A mechanistic model by which additive gene expression contributes to the maize heterotic phenotype

Theoretical models providing a biochemical basis of heterosis in plants [40] have proposed hybrid vigor to result from the presence of two alleles with differing regulatory elements, which in homozygotes would restrict growth to less than the maximum possible in a particular environment. In the heterozygous state it is suggested that they would relax their control of metabolism and growth processes, resulting in improved flux through biochemical pathways. Importantly, there is also evidence that additive gene expression impacts heterosis by balancing expression of genes in metabolic pathways; for example a more consistent, and lower variation in metabolites has been reported for heterotic maize hybrids compared with their inbred parents, presumably caused by improved metabolic flux [41]. A similar correlation between mainly additive gene expression and enhanced metabolic flux has been recorded for *Arabidopsis*[42].

A related theoretical model explaining the role of additive expression in heterosis was proposed by Springer and Stupar [43] in which additive, mid-parental gene expression levels of certain genes in the hybrid were proposed to balance out detrimental parental over- or underexpression. In support, Springer and Stupar [43] argued that many genes with optimal expression ranges are known, citing as examples genes involved in pathogen defense [44].

**Figure 6:**
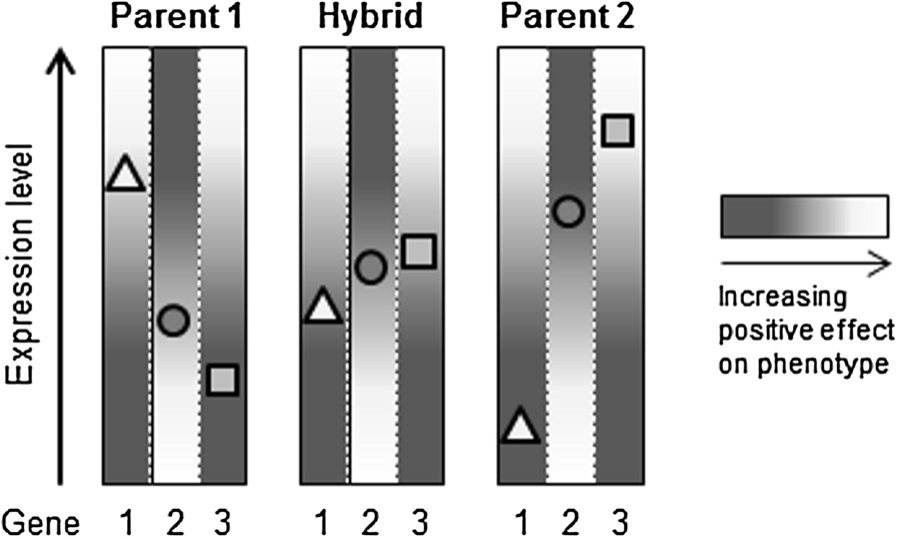
**Model explaining the influence of additively expressed, linear with heterosis-correlated genes on heterosis.** In the boxes the expression levels of three hypothetical genes in two parental inbred lines (Parent 1 and 2) and their hybrid progeny (Hybrid) are shown by triangles (gene 1), circles (gene 2) and squares (gene 3). The expression of all three genes either show a positive (genes 1 and 3) or a negative (gene 2) linear correlation with heterosis illustrated by a consistent transition from white to black; white is representing a more favorable gene expression regarding the phenotype. Additive expression levels in the hybrid are compensating for detrimental expression in one or the other parent. The cumulative effect of overall more balanced gene expression in hybrids contributes to heterosis.

We have developed a model to explain how additive gene expression impacts heterosis in maize based on the findings reported here (Figure 6). The model relies on the following precepts: 1) Our MPH-correlated genes are mainly additively expressed in hybrids, as confirmed by our findings. 2) Within a certain range, expression levels of MPH-correlated genes display either a linear positive or a linear negative correlation with heterosis, resulting in a continuous relationship between expression level and the phenotype. 3) MPH-correlated gene expression between inbred parents differs significantly. 4) The expression level of a MPH-correlated gene is more favorable in one of the parents than in the other. 5) When compared to the additive (mid-parental) value, an increase or decrease in expression level of every single gene results in a gain or loss of heterosis. 6) Additive gene expression contributes to heterosis through transcriptome-wide compensation for detrimental parental expression levels.

In our model we assume that additive expression compensates for the collective, transcriptome-wide detrimental parental expression levels of MPH-correlated genes in the hybrid and thereby contributes to the formation of heterosis. However, the highly significant, linear correlation between gene expression levels and heterosis revealed by our work suggest that an increase or decrease in the expression level of every single gene must lead to a gain or loss in the vitality of the hybrid when compared with the additive, mid-parental value.

This important finding supports a complex, multigenic model for heterosis in maize, and implies that expression level changes of genes exert a stronger influence on the performance of the hybrid than on the performance of their inbred parents. It is likely that this results from positively enhanced and altered molecular interactions in the hybrid state and may apply to both the functional capacity of two divergent alleles of the same gene [40], and to multigenic interactions. Dosage effect [45] may also be particularly important, because the proportions of single components of protein complexes, for example in signal transduction, strongly influence the efficiency of the entire complex. In the terms of quantitative genetics, the role of an altered molecular interaction in hybrids corresponds to an epistatic effect on heterosis formation [5,46,47]. Both of these mechanistic explanations are supported by the enrichment of specific functional classes of candidate genes (see above).

Thus, we assume that the linear correlation between the additive, mid-parental expression levels of particular genes and heterosis reflect both transcriptome-wide compensation of detrimental parental expression levels and intensified molecular interactions in the hybrid.

## Conclusions

We have carried out a meta-analysis approach comprising different molecular and phenotypic datasets that allow us to investigate heterosis for GY in maize. The potential role of additively expressed genes in heterosis formation is shown by their significant enrichment in known heterotic QTL for GY. Our data reveal these genes to be clustered in pericentromeric regions of the maize genome. The rapid divergence at the sequence level and the low recombination rate at pericentromeric regions explain the observed enrichment of differentially expressed alleles associated to heterosis and their assumed fixation in parental inbred lines. Based on these findings we propose a model to explain the role of additive expression in generation of heterosis for GY in maize by the compensation of fixed detrimental expression levels in parents. We anticipate that our data will aid the development of more accurate predictive molecular breeding markers for heterosis through the identification of QTL-underlying genes.

## Methods

### In silico localization of heterosis-correlated genes

In a former study we identified 1,999 genes differentially expressed between 21 parental inbred lines, which additive, mid-parental expression levels in seven-day-old seedlings were correlated (p ≤ 0.01) with MPH for GY field data of 98 hybrids [11]. In this study the 50–70 nt long oligonucleotide sequences (http://www.maizearray.org) of the 1,999 MPH-correlated were mapped against the B73 RefGen_v1 assembly (http://www.maizegdb.org) (BLASTn version 2.2.26, e-value < 0.0001). Filtering of the BLAST-results comprised elimination of all hits except the one with the lowest e-value. In the case of two or more top BLAST hits with similar e-values, all hits were eliminated to finally receive a set of oligonucleotides with a defined localization on the maize genome. Additionally, based on the identified gene IDs, all but one oligonucleotides associated to the same gene were excluded from *in silico* localization. Oligonucleotides without gene ID were excluded according to repetitive accessions (Maize Oligonucleotides Array Oligo Database v1, http://www.maizearray.org).

To analyze the distribution of the MPH-correlated genes (see Additional file 11), the B73 genome was divided into 145 segments of equal size (13,642,275 bp each). To avoid segments spanning over two chromosomes, the small remaining genome segments not large enough to build a segment on their own at the end of each chromosome were added to the previous, neighboring segment. The number of MPH-correlated genes per segment was determined.

The genomic distribution of the MPH-correlated genes was compared to a calculated gene distribution, which reflect the actual gene distribution on the B73 maize genome, for this purpose a random gene distribution was determined by using all gene ID annotated oligonucleotides of the 46k-microarray (GEO Platform accession number GPL6438) to represent a localization of genes just by chance. These oligonucleotides were mapped and filtered analogous to the MPH-correlated genes. From the 20,322 remaining oligonucleotides, random sets of oligonucleotides of the size of the mapped MPH-correlated genes (1,654 genes) were mapped in a bootstrap assay to the 145 genome segments. This allocation step of randomly chosen oligonucleotides was repeated 1,000,000 times and the average mapped oligonucleotide number per segment was determined. To analyze the possible overall deviation of the random gene distribution from the MPH-correlated genomic gene distribution, a chi-square test was performed.

Specific segments with a significant difference between the random and the MPH-correlated gene distribution were determined according to the p-value (p ≤ 0.1) of the bootstrap analysis.

### In silico QTL mapping and co-localization

In the present study, the Z_2_ (half the trait difference between pairs of backcross progenies) heterotic QTL from Schön *et al.*[4] were localized on the maize genome and were implemented in the co-localization analysis. Genetic markers directly flanking the QTL of interest on the original genetic maps of the three populations, re-analyzed by Schön *et al.*[4], were identified and physically mapped to the B73 genome (B73 RefGen_v1, http://www.maizegdb.org). The inner borders of both flanking markers defined the QTL confidence intervals. The physical positions of the mapped QTL confidence intervals were then tested for co-localization with the MPH-associated genomic segments (MPH-ASs). Bins were assigned to the B73 genome (B73 RefGen_v1) according to the core markers of the IBM2 2008 neighbor map (http://www.maizegdb.org).

### Gene function of MPH-correlated genes

Functions of the MPH-correlated genes located on the MPH-ASs were determined via the MIPS Functional Catalogue Database (FunCatDB, [18]) and by overrepresentation analysis. Overrepresented biological processes among the MPH-correlated genes located to the MPH-ASs in comparison to all genes from the 46k-microarray were assessed using the package topGO (version 1.10.1) in R (http://www.r-project.org) including the weight algorithm [48]. The annotation of the 46k-microarray was regenerated using Blast2GO [49] with default settings, resulting in an enhanced Gene Ontology (GO) annotation in which 73.6% of all genes from the 46k-microarray were successfully annotated in comparison to only 38.15% from the old annotation (http://www.maizearray.org). In Additional file 7 the resulting GO annotation of the 290 MPH-correlated genes located on MPH-ASs are shown. For the GO overrepresentation analyses, based on Fisher’s exact test, the new annotated GO terms were used. For the elimination of genes, which were represented twice or more often on the array, oligonucleotides with the same gene ID were excluded from the GO analyses. Oligonucleotides without gene ID but a repetitive accession (Maize Oligonucleotides Array Oligo Database v1, http://www.maizearray.org) were also excluded from the analysis. Genes could contribute to the overrepresentation of more than one biological process. All GO annotations of genes from overrepresented processes were manually reviewed and adjustments implemented by rerun analysis, if necessary.

### Plant material

In this study, for hybrid expression analysis, seedlings of nine diverse maize inbred lines (4 Flint and 5 Dent) and nine hybrids of the breeding program of the University of Hohenheim, Germany [12,13] were examined. Three of the four Flint inbred lines had an European Flint background (F012, F039, F047), the remaining lines had a Flint/Lancaster background (L024). The Dent inbred lines comprised two lines with an Iowa Stiff Stalk Synthetic (S028, S036) and three with an Iodent background (P024, P033, P048). The hybrids were derived from interpool-crosses (P033xF047, P048xF047, P024xF039, S036xL024, S028xL024, S028xF012, S028xF039, P024xF012) and one intrapool-cross (F012xF039). The hybrids showed varying levels of MPH for GY (see Additional file 8). Interpool hybrids with a high MPH level were P033xF047 and P048xF047, the hybrids with an intermediate MPH level were P024xF039, S028xF039 and P024xF012 and the hybrids with a weak MPH level were S036xL024 and S028xL024. GY field data was collected from field trials, for the inbred lines in 2003, 2004 and 2005 at three to five locations and for the hybrids in 2002 at six locations in Germany and measured in Mg ha^−1^ adjusted to 155 g kg^-1^ grain moisture [12]. These data were combined with field data of factorials from additional years and locations and analyzed with a mixed linear model as described in detail by Schrag *et al.*[13]. The resulting best linear unbiased predictors (BLUP) of the hybrids and lines served as phenotypic basis for this study (see Additional file 12).

In total, four biological replicates of each parental and hybrid genotype were grown under controlled conditions (25°C 16 h day, 21°C 8 h night, 70% air humidity) for seven days in a climate chamber (Percival Scientific Inc., Perry, USA). The whole seedlings were flash-frozen in liquid nitrogen.

### RNA-probe synthesis for microarrays

For the synthesis of aminoallyl RNA (aaRNA) probes, total RNA was extracted from each biological replicate (seedling) separately. Total RNA was precipitated (4M LiCl), residual genomic DNA was hydrolyzed with DNaseI (Fermentas, St. Leon-Rot, Germany) and purified with the NucleoSpin RNA Clean-up Kit (Macherey-Nagel, Düren, Germany). For first strand cDNA synthesis, 5 μg of total RNA and the Superscript II from Invitrogen (Life Technologies, Carlsbad, USA) were used. Second strand synthesis was performed using DNA Polymerase I and RNase H followed by a 5 min incubation step of T4 DNA Polymerase (Fermentas, St. Leon-Rot). Residual RNA was hydrolyzed with RNase A. In-vitro transcription was performed with the T7 RNA Polymerase (Fermentas, St. Leon-Rot, Germany) for 4h to incorporate aminoallyl-labeled UTPs (Fermentas, St. Leon-Rot, Germany). Finally, residual DNA was degraded with DNase I (Fermentas, St. Leon-Rot, Germany).

Synthesized aaRNA was coupled with fluorescence dyes Cy3 or Cy5 (GE Healthcare, Chalfont St. Giles, UK). The RNeasy MinElute Kit (Qiagen, Hilden, Germany) was used for purification and removal of unbound dye.

### Microarray analysis

Expression analysis of MPH-correlated genes was performed with a custom-made 1.5k-microarray (GEO Platform accession number GPL17936) with 732 genes printed in duplicates. Based on our previous study [11], the probes were selected from the 46k-microarray (University of Arizona). In the course of this former study we identified genes differentially expressed between 21 parental inbred lines, which additive, mid-parental expression levels in seven-day-old seedlings were correlated with GY and/or grain dry matter content field data of 98 hybrids. The genes of interest for this heterosis-study and which we consider for hybrid expression analysis comprise 345 MPH for GY-correlated genes and 174 non-correlated genes. The 345 genes comprised the 200 most significantly MPH-correlated genes and 145 genes with additional overlapping correlations to hybrid performance and/or grain dry matter content for GY. The latter group comprised non-correlated genes, which belonged to the same top 6 biological processes that were found to be enriched among the HP for GY-correlated genes [11].

For the custom-made 1.5k-microarray we used oligonucleotide sequences (50-70nt) of the maize oligonucleotide array project (University of Arizona, USA; http://www.maizearray.org). The oligonucleotides were synthesized by Ocimum Biosolutions (Ijsselstein, Netherlands) and printed on poly-L-lysine coated glass slides with a Microgrid II printer (BioRobotics, Boston, USA). After probe labeling and hybridization, following the protocol of the maize oligonucleotide array project (http://www.maizearay.org), the microarrays were scanned (Applied Precision ArrayWorx Scanner, Applied Precision Inc., USA) and analyzed using the GenePix Pro 4.0 software (Molecular Devices, Sunnyvale, USA). Hybridizations between every inbred pair and its corresponding hybrid were conducted four times, one time with every set of biological replicates. The hybridization scheme is depicted in Additional file 13. Between biological replicates the dye (Cy3 or Cy5, GE Healthcare, Chalfont St. Giles, UK) used for hybridization was alternated for reduction of systematic bias. In total, 105 hybridizations were performed. Data normalization was performed using the LIMMA package (version 2.9) from Bioconductor [50]. For assessing differential expression, the *normexp* background correction method with the parameter offset = 50 was applied to each array and resulted in strictly positive adjusted intensities. The adjusted signal intensities were normalized using a two-step normalization: weighted print-tip group loess normalization of log-ratios (within-array normalization) followed by quantile normalization of average log-intensities of two channels (between-array normalization). A series of positive control probes were given double weight to assist the normalization process.

### Differentially expressed genes and characterization of hybrid expression

Differential expression between genotypes was assessed within each of the nine hybrid-inbred triplets by one-way ANOVA. A Benjamini and Hochberg correction with a false discovery rate (FDR) of 0.15 was applied. We only considered genes with gene expression data from all four biological replicates, with an expression fold-change of at least 1.3 between any two of the three genotypes and with a minimum expression level (log2) of 8.0 in at least one genotype.

To discriminate between additive (mid-parental) and non-additive expression, the differentially expressed genes were further analyzed, according to Stupar *et al.*[51] for comparability of the results between studies. For that matter t-tests (p ≤ 0.05, two-tailed, paired) between the hybrid expression levels of the biological replicates and the average expression levels from the replicates of both parental inbred lines were conducted.

Hybrid gene expression was declared low parent-like (LPL) or high parent-like (HPL) if no significant difference was detected between the biological replicates of the hybrid and the low parent or the high parent, respectively (*t*-test, two-tailed, paired, p ≤ 0.05). For the identification of hybrid expression levels significantly higher (above high parent, AHP) or lower (below low parent, BLP) than both parents a one-sided, paired *t*-test (p ≤ 0.05) was conducted. The remaining differential non-additively expressed genes were declared as expressed between both parents.

### Availability of supporting data

The custom-made 1.5k-microarray has been deposited in NCBI’s Gene Expression Omnibus [52] and is accessible through GEO Platform accession number GPL17936.

The data discussed in this publication are accessible through GEO Series accession number GSE52411 (http://www.ncbi.nlm.nih.gov/geo/query/acc.cgi?acc=GSE52411).

## Abbreviations

BPH: Best-parent heterosis; GO: Gene ontology; GY: Grain yield; HP: Hybrid performance; MPH: Mid-parent heterosis; MPH-AS(s): Mid-parent heterosis of grain yield associated segment(s); QTL: Quantitative trait locus/loci.

## Competing interest

The authors declare no competing financial interests.

## Authors’ contributions

AT wrote the paper, interpreted the results, performed the *in silico* and statistical analyses, and conducted the microarray experiments, including the cultivation of the plants. JF conducted the microarray normalization and helped to write the paper. FS performed the oligonucleotide mapping, the bootstrap analysis, and helped to write the paper. RTG was involved in the interpretation of the results and helped to write the paper. TAS and AEM gathered and analyzed the field data. HP, MF, AEM, and SS contributed to the statistical analyses and to the writing of the paper. AT and SS devised and planned the study. All authors read and approved the final manuscript.

## Supplementary Material

Additional file 1 **Field data of the 21 inbred parents and their 98 corresponding hybrids.** Table gives the estimates (EST) and standard errors (SE) of hybrid performance (HP) and per se (PS) performance of grain yield (in Mg ha^−1^ adjusted to 155 g kg^−1^ grain moisture) of the corresponding parental lines, which were obtained on basis of the corresponding BLUPs (best linear unbiased prediction) and SEs. Dividing mid-parent heterosis (MPH) by the mid-parent performance provided the relative MPH (MPHr). Click here for file

Additional file 2 **Results from bootstrap analysis for enrichment/depletion of MPH for GY-correlated genes in genomic segments.** Table of the bootstrap analysis showing the location of the genomic segments, the count number of MPH for GY- and non-correlated genes, and the probability of enrichment or depletion of the MPH for GY-correlated genes. Click here for file

Additional file 3 **MPH-ASs with a significantly increased number of MPH for GY-correlated genes.** The position of the 15 MPH-associated genomic segments (MPH-AS[chromosome.segment]) with an elevated number of MPH for GY-correlated genes is depicted by the chromosome, the Bin and the physical position. In addition the numbers of MPH-correlated genes within the segments and the p-values of the bootstrap assay are given. Click here for file

Additional file 4 **Statistical comparison of MPH for GY-correlated differential gene expression.** The table shows the p-values for differential expression of all MPH-correlated genes between the 21 parental inbred lines from Thiemann *et al.* 2010 [11]. The genes are differentiated between those localized and not localized on MPH-ASs. Also given are the p-value of the *t*-test and the average amount of differential expression per gene together with the standard deviations (SD) and standard errors (SE). Click here for file

Additional file 5 **Heterotic QTL for GY from Schön *et al. ***[4]**with a dominance effect (Z**_**2**_**) and their co-localizing MPH-ASs.** Shown are the positions of heterotic QTL for GY from three populations re-analyzed by Schön *et al.*[4] with significant dominance effects and a position on all ten chromosomes. The three populations originated from studies of Stuber *et al.*[14] (Pop1), Lu *et al.*[15] (Pop2) and Frascaroli *et al.*[16] (Pop3). The chromosome, the Bin, the flanking markers and the physical position describe the position of the QTL. Interrogation marks indicate genetic markers with unknown physical position. In the right column the MPH-ASs(chromosome.segment) the QTL co-localize with are shown. Click here for file

Additional file 6 **Blast2GO annotation of the MPH for GY-correlated genes located on MPH-ASs.** Table of the 290 MPH-correlated oligos located on MPH-ASs with their annotation received from Blast2GO analysis, including BLASTx hits and their GO annotation (Annotation from GEO platform accession number GPL17936). Click here for file

Additional file 7 **Overrepresented GO terms for biological processes of genes correlated with MPH for GY and located on MPH-ASs.** GO terms for biological processes (BP) significantly (p ≤ 0.05) overrepresented among genes correlated with MPH for GY and located on MPH-ASs. The number of genes belonging to each BP among the genes correlated with MPH for GY [Genes (no.)] and p-values (weight algorithm) indicating the significance of the overrepresentation are given. Click here for file

Additional file 8 **Field data of the inbred parents and their eight corresponding interpool hybrids.** Given are the field data from the eight analyzed interpool-crosses, including the performance for GY measured in Mg ha^−1^ adjusted to 155 g kg^−1^ grain moisture. Estimates (EST) and standard errors (SE) of hybrid performance (HP) and per se (PS) performance of their corresponding parental lines were obtained on basis of the corresponding BLUPs (best linear unbiased prediction) and SEs. Dividing mid-parent heterosis (MPH) by the mid-parent performance provided the relative MPH (MPHr). Click here for file

Additional file 9 **One-way ANOVA (FDR < 0.15) results for the identification of differentially expressed genes.** Table of genes used in one-way ANOVA (FDR < 0.15) after filtering process (see Methods). Given are the average expression values of each gene for each inbred and their corresponding hybrid. Genes still significant after Benjamini and Hochberg correction are marked with a “1” and non-significant genes with a “0”. Click here for file

Additional file 10 ***T*-test results for the identification of additive and non-additive expression pattern.** Table of differentially expressed genes used in two-sided paired t-tests (p < 0.05). Given are the average expression values of each differentially expressed gene of the calculated mid-parental value and the corresponding hybrid. In the last column the types of non-additive expression are given. Click here for file

Additional file 11 ***In silico *localization of the MPH for GY-correlated genes located on MPH-ASs.** Table of the 290 MPH-correlated oligos located on MPH-ASs with their respective oligo sequence and annotation received from BLASTn (2.2.26+) analysis including Gene-IDs, Chromosome positons and BLAST e-values. Click here for file

Additional file 12 **Estimates of variance components (VC) and standard errors (SE) from field data.** Estimates of variance components (VC) and their standard errors (SE) of general combining ability (GCA) of flint (f) and dent (d) parents and specific combining ability (SCA) together with their interactions with locations (LOC) and years (YEAR). Biometrical analyses comprised phenotypic data (grain yield in Mg ha^−1^) of 400 crosses and their 79 parental inbred lines, of which the crosses considered in this study were a subset. Click here for file

Additional file 13 **Microarray (1.5k-microarray) hybridization scheme.** The white and black circles indicate the dent and the flint inbred lines, respectively. The grey circles represent the corresponding hybrids. The lines connecting the circles demonstrate hybridizations. The hybridization scheme was conducted in total four times, each time with a different set of biological replicates and with alternating fluorescent dye labeling. Click here for file
