# Supplementary material for: Genome-wide meta-analysis of maize heterosis reveals the potential role of additive gene expression at pericentromeric loci
Source: BMC Plant Biol. 2014 Apr 2;14:88. doi: 10.1186/1471-2229-14-88 (PMC4234143; doi:10.1186/1471-2229-14-88)
Supplement: Additional file 3 — MPH-ASs with a significantly increased number of MPH for GY-correlated genes. The position of the 15 MPH-associated genomic segments (MPH-AS[chromosome.segment]) with an elevated number of MPH for GY-correlated genes is depicted by the chromosome, the Bin and the physical position. In addition the numbers of MPH-correlated genes within the segments and the p-values of the bootstrap assay are given. [file 1471-2229-14-88-S3.doc]

**Additional file 3 MPH-ASs with a significantly increased number of MPH for GY-correlated genes.**

| MPH-associated segments (MPH-ASs) | Chromosome | Bin (B73 GefGen_v1) | Physical position (B73 GefGen_v1) | Number of MPH for GY-correlated genes | Bootstrapping p-values of MPH-ASs |
| --- | --- | --- | --- | --- | --- |
| MPH-AS1.1 | 1 | 1.00 - 1.02 | 1 -  13,642,275 | 30 | 0.049 |
| MPH-AS1.8 | 1 | 1.05 | 95,495,926 -  109,138,200 | 11 | 0.019 |
| MPH-AS1.9 | 1 | 1.05 | 109,138,201 -  122,780,475 | 7 | 0.0775 |
| MPH-AS1.12 | 1 | 1.05 | 150,065,026 -  163,707,300 | 10 | 0.0109 |
| MPH-AS1.14 | 1 | 1.06 | 177,349,576 -  190,991,850 | 19 | 0.0194 |
| MPH-AS1.15 | 1 | 1.06 - 1.07 | 190,991,851 - 204,634,125 | 21 | 0.029 |
| MPH-AS2.13 | 2 | 2.06 - 2.07 | 163,707,301 - 177,349,575 | 15 | 0.093 |
| MPH-AS2.16 | 2 | 2.08 | 204,634,126 - 218,276,400 | 28 | 0.0762 |
| MPH-AS3.9 | 3 | 3.04 - 3.05 | 109,138,201 -  122,780,475 | 9 | 0.0737 |
| MPH-AS3.10 | 3 | 3.05 | 122,780,476 -  136,422,750 | 17 | 0.0151 |
| MPH-AS5.3 | 5 | 5.03 | 27,284,551 -  40,926,825 | 19 | 0.0082 |
| MPH-AS8.3 | 8 | 8.03 | 27,284,551 -  40,926,825 | 10 | 0.0889 |
| MPH-AS8.5 | 8 | 8.03 | 54,569,101 -  68,211,375 | 11 | 0.0399 |
| MPH-AS8.9 | 8 | 8.04 - 8.05 | 109,138,201 -  122,780,475 | 22 | 0.0028 |
| MPH-AS8.12 | 8 | 8.06 - 8.09 | 150,065,026 -  174,515,299 | 61 | 0.0091 |

The position of the 15 MPH-associated genomic segments (MPH-AS[chromosome.segment]) with an elevated number of MPH for GY-correlated genes is depicted by the chromosome, the Bin and the physical position. In addition the numbers of MPH-correlated genes within the segments and the p-values of the bootstrap assay are given.
