# Supplementary material for: Genome-wide meta-analysis of maize heterosis reveals the potential role of additive gene expression at pericentromeric loci
Source: BMC Plant Biol. 2014 Apr 2;14:88. doi: 10.1186/1471-2229-14-88 (PMC4234143; doi:10.1186/1471-2229-14-88)
Supplement: Additional file 5 — Heterotic QTL for GY from Schön et al. [4]with a dominance effect (Z2) and their co-localizing MPH-ASs. Shown are the positions of heterotic QTL for GY from three populations re-analyzed by Schön et al.[4] with significant dominance effects and a position on all ten chromosomes. The three populations originated from studies of Stuber et al.[14] (Pop1), Lu et al.[15] (Pop2) and Frascaroli et al.[16] (Pop3). The chromosome, the Bin, the flanking markers and the physical position describe the position of the QTL. Interrogation marks indicate genetic markers with unknown physical position. In the right column the MPH-ASs(chromosome.segment) the QTL co-localize with are shown. [file 1471-2229-14-88-S5.doc]

**Additional file 5 Heterotic QTL for GY from Schön *et al.* [4] with a dominance effect (Z_2_**) and their co-localizing MPH-ASs.

| Population | Chromosome | Bin | Marker  interval | Physical pos. of QTL (B73 GefGen_v1) | Co-localizing MPH-ASs(chr.segments) |
| --- | --- | --- | --- | --- | --- |
| Pop1 | 1 | 1.06 - 1.07 | npi429 -amp1 | 184,626,675 -224,185,366 | MPH-AS1.14; 1.15 |
| Pop1 | 2 | 2.07 | npi297 -npi456 | 173,769,221 -175,485,839 | MPH-AS2.13 |
| Pop1 | 3 | 3.07 -3.09 | [npi212b](http://www.maizegdb.org/cgi-bin/displaylocusrecord.cgi?id=12691) - a1 | 196,899,793 -214,729,506 | - |
| Pop1 | 4 | 4.05 | bni15.45 -npi267 | 76,669,629 - 139,911,213 | - |
| Pop1 | 4 | 4.08 -4.09 | npi104 - npi317 | 198,562,221 - 223,858,689 | - |
| Pop1 | 5 | 5.04 | npi449a - amp3 | 154,598,241 - 168,236,518 | - |
| Pop1 | 7 | 7.03 | npi394 - npi283a | 127,776,190 - 142,205,407 | - |
| Pop1 | 8 | 8.03 - 8.06 | bnl1.45 - npi425b | 100,564,148 - 163,151,864 | MPH-AS8.9; 8.12 |
| Pop1 | 10 | 1.00 | npi366 - npi371c | 769,324 - 1,523,962 | - |
| Pop1 | 10 | 10.03 - 10.04 | glu1 -  pi264 | 34,240,639 -118,755,957 | - |
| Pop2 | 1 | 1.02 -  1.03 | bnlg1007 -  bnlg2204 | 26,829,500 -  44,691,894 | - |
| Pop2 | 1 | 1.06 - ? | bnlg2057 -asg057 | 184,862,647 -? | MPH-AS1.14 |
| Pop2 | 1 | 1.09 - ? | phi011 -dup12 | 257,414,871 - ? | - |
| Pop2 | 2 | 2.03 | bnlg1537 -bnlg2248 | 17,268,746 -22,984,734 | - |
| Pop2 | 3 | 3.04 | bnlg1638 -phi053 | 24,517,896 -122,407,114 | MPH-AS3.9 |
| Pop2 | 3 | 3.06 | bnlg1951 -bnlg1160 | 183,560,763 -185,804,708 | - |
| Pop2 | 5 | 5.04 - 5.06 | bnlg1208 - umc126a | 150,043,318 -190,081,611 | - |
| Pop2 | 6 | 6.01 | bnlg426 - bnlg1867 | 16,248,296 - 19,422,410 | - |
| Pop2 | 7 | 7.02 | bnlg1792 - bnlg1808 | 74,186,347 - 119,302,872 | - |
| Pop2 | 7 | 7.03 - 7.04 | bnlg339 - umc1029 | 131,455,352 - 155,663,681 | - |
| Pop2 | 8 | 8.03 - 8.05 | bnlg2082 - bnlg2181 | 36,053,157 - 132,424,196 | MPH-AS8.3; 8.5; 8.9 |
| Pop2 | 9 | 9.04 | bnlg1714 - bnlg1209 | 105,340,263 - 106,411,754 | - |
| Pop2 | 10 | 10.02 - ? | phi059 - ? | 8,398,782 - ? | - |
| Pop3 | 1 | 1.04 -1.06 | dupssr26 -umc1035 | 74,038,984 -195,053,868 | MPH-AS1.8; 1.9; 1.12; 1.14;1.15 |
| Pop3 | 1 | 1.04 -  1.06 | bnlg1556 -bnlg1025 | 207,769,856 -223,005,347 | - |
| Pop3 | 2 | 1.04 -  1.06 | phi127 -mmc0271 | 186,885,023 -193,637,911 | MPH-AS2.13 |
| Pop3 | 3 | 1.04 -  1.06 | XO6755 -dupssr23 | ? -166,783,888 | - |
| Pop3 | 4 | 4.01 - 4.03 | nc135 - umc2176 | 2,200,453 - 17,476,619 | - |
| Pop3 | 4 | 4.01 - 4.09 | umc1011 - bnlg589 | 998,247 - 242,257,375 | - |
| Pop3 | 6 | 6.00 - 6.01 | bnlg161b - bnlg1371 | 3,577,325 - 15,912,003 | - |
| Pop3 | 6 | 6.07 | phi070 - dupssr15 | 162,069,867 - 162,653,440 | - |
| Pop3 | 8 | 8.03 | umc1904 - bnl9.08 | 68,656,379 - 72,402,982 | - |
| Pop3 | 8 | 8.03 - 8.05 | phi121 - bnlg666 | 104,020,029 - 132,424,196 | MPH-AS8.9 |
| Pop3 | 9 | 9.04 - 9.05 | bnlg1209 - umc95 | 106,544,469 - 122,584,372 | - |
| Pop3 | 10 | 10.01 - 10.03 | bnlg1451 - umc2016 | 4,401,330 - 62,062,609 | - |
| Pop3 | 10 | 10.04 | umc64a - umc2003 | 85,631,734 - 126,827,195 | - |

Shown are the positions of heterotic QTL for GY from three populations re-analyzed by Schön *et al.* [4] with significant dominance effects and a position on all ten chromosomes. The three populations originated from studies of Stuber *et al.* [14] (Pop1), Lu *et al.* [15] (Pop2) and Frascaroli *et al.* [16] (Pop3). The chromosome, the Bin, the flanking markers and the physical position describe the position of the QTL. Interrogation marks indicate genetic markers with unknown physical position. In the right column the MPH-ASs(chromosome.segment) the QTL co-localize with are shown.
