# Supplementary material for: Genome-wide meta-analysis of maize heterosis reveals the potential role of additive gene expression at pericentromeric loci
Source: BMC Plant Biol. 2014 Apr 2;14:88. doi: 10.1186/1471-2229-14-88 (PMC4234143; doi:10.1186/1471-2229-14-88)
Supplement: Additional file 7 — Overrepresented GO terms for biological processes of genes correlated with MPH for GY and located on MPH-ASs. GO terms for biological processes (BP) significantly (p ≤ 0.05) overrepresented among genes correlated with MPH for GY and located on MPH-ASs. The number of genes belonging to each BP among the genes correlated with MPH for GY [Genes (no.)] and p-values (weight algorithm) indicating the significance of the overrepresentation are given. [file 1471-2229-14-88-S7.doc]

**Additional file 7 Overrepresented GO terms for biological processes of genes correlated with MPH for GY and located on MPH-ASs.**

| GO term | Genes (no.) | p-value |
| --- | --- | --- |
| DNA-dependent DNA replication initiation (GO:0006270) | 3 | 0.002 |
| cold acclimation (GO:0009631) | 2 | 0.0051 |
| nickel cation transport (GO:0015675) | 1 | 0.0091 |
| transcription from RNA polymerase I promoter (GO:0006360) | 1 | 0.0181 |
| allantoin transport (GO:0015720) | 1 | 0.0181 |
| peptidyl-arginine methylation, to asymmetrical-dimethyl arginine (GO:0019919) | 1 | 0.0181 |
| positive regulation of DNA endoreduplication (GO:0032877) | 1 | 0.0181 |
| regulation of DNA repair (GO:0006282) | 1 | 0.027 |
| cholesterol biosynthetic process (GO:0006695) | 1 | 0.027 |
| non-photochemical quenching (GO:0010196) | 1 | 0.027 |
| small GTPase mediated signal transduction (GO:0007264) | 5 | 0.0277 |
| oligopeptide transport (GO:0006857) | 3 | 0.0279 |
| amino sugar metabolic process (GO:0006040) | 1 | 0.0359 |
| response to symbiotic fungus (GO:0009610) | 1 | 0.0447 |
| response to lead ion (GO:0010288) | 1 | 0.0447 |
| malate transport (GO:0015743) | 1 | 0.0447 |

GO terms for biological processes (BP) significantly (p ≤ 0.05) overrepresented among genes correlated with MPH for GY and located on MPH-ASs. The number of genes belonging to each BP among the genes correlated with MPH for GY [Genes (no.)] and p-values (weight algorithm) indicating the significance of the overrepresentation are given.
