# Supplementary material for: Genome-wide meta-analysis of maize heterosis reveals the potential role of additive gene expression at pericentromeric loci
Source: BMC Plant Biol. 2014 Apr 2;14:88. doi: 10.1186/1471-2229-14-88 (PMC4234143; doi:10.1186/1471-2229-14-88)
Supplement: Additional file 12 — Estimates of variance components (VC) and standard errors (SE) from field data. Estimates of variance components (VC) and their standard errors (SE) of general combining ability (GCA) of flint (f) and dent (d) parents and specific combining ability (SCA) together with their interactions with locations (LOC) and years (YEAR). Biometrical analyses comprised phenotypic data (grain yield in Mg ha−1) of 400 crosses and their 79 parental inbred lines, of which the crosses considered in this study were a subset. [file 1471-2229-14-88-S12.doc]

| **Additional file 12** **Estimates of variance components (VC) and standard errors (SE) from field data.** | | |
| --- | --- | --- |
| Name | VC | SE |
| GCAd | 0.221 | 0.064 |
| GCAf | 0.151 | 0.056 |
| SCA | 0.013 | 0.009 |
| GCAd x LOC | -0.003 | 0.002 |
| GCAf x LOC | 0.004 | 0.01 |
| SCA x LOC | 0.001 | 0.013 |
| GCAd x YEAR | 0.018 | 0.016 |
| GCAf x YEAR | 0.044 | 0.03 |
| SCA x YEAR | 0.066 | 0.01 |
| GCAd x LOC x YEAR | 0.102 | 0.012 |
| GCAf x LOC x YEAR | 0.174 | 0.025 |
| SCA x LOC x YEAR | 0.06 | 0.016 |

Estimates of variance components (VC) and their standard errors (SE) of general combining ability (GCA) of flint (f) and dent (d) parents and specific combining ability (SCA) together with their interactions with locations (LOC) and years (YEAR). Biometrical analyses comprised phenotypic data (grain yield in Mg ha^-1^) of 400 crosses and their 79 parental inbred lines, of which the crosses considered in this study were a subset.
