# Supplementary figures and images for: Genome-wide meta-analysis of maize heterosis reveals the potential role of additive gene expression at pericentromeric loci
Source: BMC Plant Biol. 2014 Apr 2;14:88. doi: 10.1186/1471-2229-14-88 (PMC4234143; doi:10.1186/1471-2229-14-88)

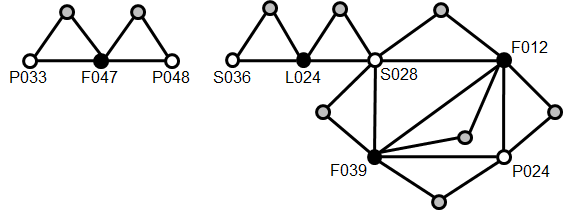

Supplement: Additional file 13 — Microarray (1.5k-microarray) hybridization scheme. The white and black circles indicate the dent and the flint inbred lines, respectively. The grey circles represent the corresponding hybrids. The lines connecting the circles demonstrate hybridizations. The hybridization scheme was conducted in total four times, each time with a different set of biological replicates and with alternating fluorescent dye labeling. [file 1471-2229-14-88-S13.tiff]
